# Period doubling as an indicator for ecosystem sensitivity to climate extremes

**DOI:** 10.1038/s41598-019-56080-z

**Published:** 2019-12-20

**Authors:** Omer Tzuk, Sangeeta Rani Ujjwal, Cristian Fernandez-Oto, Merav Seifan, Ehud Meron

**Affiliations:** 1 Department of Physics, Ben-Gurion University of the Negev, Beer-Sheva, 8410501 Israel; 2 Department of Solar Energy and Environmental Physics, SIDEER, Blaustein Institutes for Desert Research, Ben-Gurion University of the Negev, Sede Boqer Campus, Midreshet Ben-Gurion, 8499000 Israel; 3 Complex Systems Group, Facultad de Ingeniería y Ciencias Aplicadas, Universidad de los Andes, Av. Mon. Alvaro del Portillo 12.455, Santiago, Chile; 4 Mitrani Department of Desert Ecology, SIDEER, Blaustein Institutes for Desert Research, Ben-Gurion University of the Negev, Sede Boqer Campus, Midreshet Ben-Gurion, 8499000 Israel

**Keywords:** Climate-change ecology, Ecological modelling, Nonlinear phenomena, Phase transitions and critical phenomena

## Abstract

The predictions for a warmer and drier climate and for increased likelihood of climate extremes raise high concerns about the possible collapse of dryland ecosystems, and about the formation of new drylands where native species are less tolerant to water stress. Using a dryland-vegetation model for plant species that display different tradeoffs between fast growth and tolerance to droughts, we find that ecosystems subjected to strong seasonal variability, typical for drylands, exhibit a temporal period-doubling route to chaos that results in early collapse to bare soil. We further find that fast-growing plants go through period doubling sooner and span wider chaotic ranges than stress-tolerant plants. We propose the detection of period-doubling signatures in power spectra as early indicators of ecosystem collapse that outperform existing indicators in their ability to warn against climate extremes and capture the heightened vulnerability of newly-formed drylands. The proposed indicator is expected to apply to other types of ecosystems, such as consumer–resource and predator–prey systems. We conclude by delineating the conditions ecosystems should meet in order for the proposed indicator to apply.

## Introduction

The increasing number of studies associating climate extremes, such as heat waves, floods and droughts, with global warming, and the projections for more frequent and intense events in the future^1–4^, are matters of much concern among decision makers^5^. The concerns stem, in part, from the implied feasibility of abrupt and irreversible ecosystem responses to such events. This response form, often termed a “regime shift”^6^, is shared by many ecosystems, where positive feedbacks between ecological processes result in multiplicity of stable states. Regime shifts, which amount to transitions between alternative stable states, can have far-reaching consequences in terms of ecosystem function and services. In drylands, which occupy 40% of the terrestrial Earth surface, severe droughts may result in abrupt desertification and loss of ecosystem services, such as crop and livestock production, flood control, soil formation and others^7^. These concerns are further increased by recent predictions of accelerated dryland expansion and the concomitant increase in ecosystems that are sensitive to climate extremes^8^.

Numerous studies have been devoted to the development and empirical testing of early warning signals (EWS) to impending regime shifts^9–11^. Most of these signals are based on a generic property of dynamical systems near tipping points (saddle-node bifurcations), namely, the slow decay of perturbations or “critical slowing down”. Several EWS utilizing this effect have been proposed and studied, including the increase of temporal autocorrelation and variance^12,13^ and of skewness of time-series distributions^14^. Implicit in this approach is the assumption of slowly drifting environmental conditions that drive ecosystems to the immediate neighborhoods of tipping points, where these indicators become relevant. This assumption does not apply to extreme events that can drive ecosystem collapse even further from tipping points^15^. Vulnerability to such events should be sought, instead, in identifiable processes that precede tipping points or other collapse thresholds, as we propose in this work.

In drylands, ecosystem collapse can be preceded by self-organization in spatial vegetation patterns^16–18^. This is a population-level response to water stress that involves the creation of water-contributing bare-soil areas, and improves the resilience to droughts relative to ecosystems that do not show spatial patterning. In a sense, it is a sign of ecosystem health, indicating a successful employment of a process that culminates in an additional source of water to vegetation patches–water transport from adjacent bare-soil areas^19,20^. In this study we focus on another possible type of processes that may precede ecosystem collapse in drylands, associated with temporal chaotic oscillations rather than with spatial patterns. Unlike spatial patterning, this process results in *early* ecosystem collapse. Studying this process may therefore be crucial for assessing vulnerability to climate extremes.

A key point in our study is the consideration of seasonal periodicity of environmental conditions, such as temperature and rainfall. This generic aspect of most ecosystems is particularly significant when intrinsic time scales, often associated with competition over limiting resources, are comparable to the seasonal periodicity^21,22^. Intrinsic time scales often appear in the form of oscillatory decay of disturbances about a stable equilibrium state, which implies the existence of damped oscillatory modes. When the time scales of these modes match the periodicity of an external forcing, sustained large-amplitude oscillations can result^23^. This effect has been demonstrated recently in a long-term empirical study of cyclic species succession in a rocky intertidal community^24,25^. Complementary model studies^24^ with increasing seasonality strength have shown damped oscillations turning into periodic oscillations and further on into chaotic oscillations, as observed empirically. However, this and earlier studies of seasonally forced ecosystems^26^ have not addressed the question of possible early ecosystem collapse and a timely prediction of such collapse as a result of a climate extreme.

**Figure 1:**
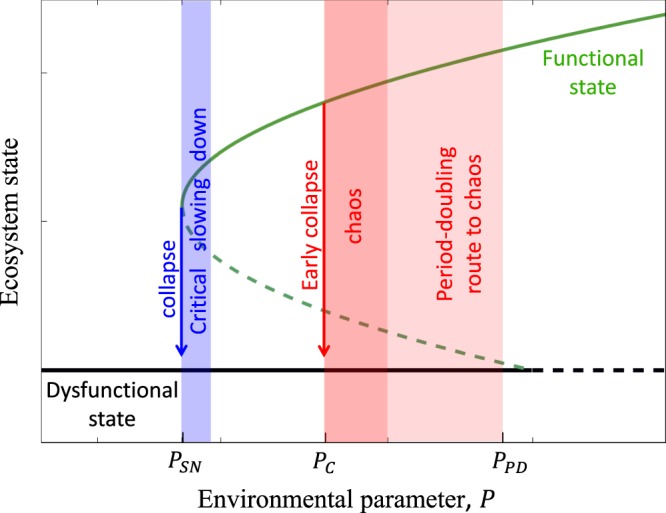
A schematic illustration of early ecosystem collapse induced by period-doubling route to chaos in seasonally forced systems. Items in blue color represent the prevailing paradigm of ecosystem collapse across a tipping point, and the narrow range of critical slowing down that precedes the collapse in which existing early-warning signals apply. Items in red represent generic dynamical behaviors of seasonally forced systems. They include a cascade of period-doubling bifurcations, chaotic dynamics and early ecosystem collapse. The label *P*_*SN*_ represents a threshold of an environmental parameter, such as precipitation, at which the functional state that describes resonant oscillations ceases to exist, the label *P*_*PD*_ represents the threshold at which the oscillation period of the functional state doubles, and the label *P*_*C*_ represents the threshold at which collapse to a dysfunctional state occurs.

In this paper we show that the common view of desertification as a tipping-point phenomenon^6^ is not necessarily valid when seasonal forcing is taken into account. Using a two-soil-layer model^27^ that better accounts for the soil-water dynamics than traditional vegetation models^28,29^, we show that seasonality can drive damped oscillatory modes into chaotic oscillations and early collapse to bare soil. The chaotic dynamics, in turn, are preceded by a cascade of period doubling bifurcations, which provide the identifiable process needed for early warning against climate extremes, as Fig. 1 schematically illustrates. We suggest the detection of period-doubling signatures in power spectra^30^ as an indicator for ecosystem vulnerability to extreme droughts, and study its applicability also in the presence of rainfall stochasticity. We further consider plant functional groups that exhibit different tradeoffs between fast growth and tolerance to drought, in order to distinguish between plant species with different evolutionary histories of water stress. This will allow us to compare the responses of species with different levels of adaptation to drought and hence to study the peculiarities of newly formed drylands.

## Results

### Vegetation model

We consider the dynamics of a water-limited plant population in a homogeneous flat terrain, assuming the vegetation is spatially uniform, that is, pattern-forming feedbacks are too weak to induce spatial instabilities^20^. An important aspect of the model that we use is the consideration of the soil depth dimension^27^, which allows us to account for the slower soil-water dynamics associated with water percolation into deeper root zones. The strict time-scale separation in simpler models that do not account for the soil depth dimension, such as in Klausmeier model^31^, does not allow for the oscillatory phenomena that can be observed in our model. The model consists of a single biomass variable, *B*, that represents the above-ground biomass density of a plant species, and two soil-water variables, *s*_1_ and *s*_2_, representing the relative soil-moisture levels in a top soil layer and in a deeper soil layer, respectively. For simplicity, we assume high infiltration rates of surface water into the soil, which allows us to disregard overland water dynamics^19^. We also assume that water loss by evaporation occurs from the top soil layer only, while water uptake by the plant’s roots is from the deep soil layer only. The modeling of these processes also capture two distinct positive feedbacks between biomass and water that lead to bistability-precipitation ranges of vegetation and bare-soil states. The first is associated with reduced evaporation by shading in areas of growing vegetation, and the second is related to the root-to-shoot ratio and the increased water uptake of grown vegetation.

In order to study the different responses to seasonal forcing of dryland ecosystems with long and short histories of water stress, we adopt a trait-based approach. We focus on two functional traits: the plant growth rate, Λ, and the attenuation, *M*_*s*_, of the mortality rate by the activation of a survival mechanism as the deep-soil moisture *s*_2_ drops below a characteristic value. The survival mechanism may be attributed to a physiological shift in individual plants from fast resource usage and new tissue building (including a high rate of stomatal activity) toward a more efficient maintenance activity whose goal is to invest mainly in differentiation processes and transpiration^32^. We further assume that there is a tradeoff between investment in growth and investment in tolerance to water stress^33^, and quantify this tradeoff by a tradeoff parameter 0 ≤ *χ* ≤ 1, such that *χ* = 0 represents a functional group of plants investing mostly in fast growth (short water-stress history), and *χ* = 1 represents a functional group investing mostly in tolerance (long water-stress history).

Seasonal forcing is introduced by modulating the precipitation parameter as:

1 $$P(t)={P}_{0}(1+a\,\cos (2\pi \frac{t}{T})),$$

where *P*_0_ is the mean annual rainfall, taken to be constant or stochastic in time, *a* is the amplitude of the periodic variations, and *T* = 1 year. Focusing on questions of ecosystem collapse associated with seasonal forcing, we choose the unforced system (*a* = 0) to show a bistability precipitation-range of bare soil and steady vegetation, within which the stable vegetation state has damped oscillatory modes (oscillatory decay of perturbations). We refer the reader to the Methods section for additional information about the model.

### Response to seasonal forcing

**Figure 2:**
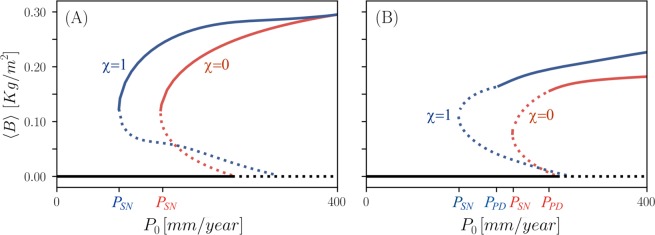
Bifurcation diagrams of resonant vegetation oscillations under weak (**A**) and strong (**B**) seasonal forcing, obtain from Eq. (2). Horizontal and vertical axes represent precipitation rate and annual biomass average, respectively. Solid (dashed) lines represent stable (unstable) solutions. The blue curves describe 1:1 oscillating-vegetation solutions of the functional group *χ* = 1 (highest investment in tolerance to water stress), while the red curves describe similar solutions of the functional group *χ* = 0 (highest investment in fast growth). The black lines describe the bare-soil solution, which remains stable up to the bifurcation points with the oscillating-vegetation solutions. The label *P*_*SN*_ denotes the saddle-node bifurcation at which the 1:1 oscillating-vegetation solutions cease to exist, which depends on the seasonality strength *a*. The label *P*_*PD*_ denotes a period-doubling instability of 1:1 oscillating-vegetation solutions. Parameters are as in Table 1 and *a* = 0.01 (**A**), *a* = 1.0 (**B**).

When the seasonal forcing is sufficiently weak all functional groups, 0 ≤ *χ* ≤ 1, respond similarly by small-amplitude resonant oscillations with periods equal to the annual seasonal periodicity, hereafter a 1:1 response. All functional groups also show bistability of bare-soil and vegetation states, but the range of bistability depends on the specific functional group, as the bifurcation diagram in Fig. 2a shows. Vegetation states of plant species investing in growth (low *χ*) are the first to appear from the bare-soil state upon increasing the mean annual precipitation *P*_0_ (red line), but are also the first to disappear upon decreasing *P*_0_ from high values, in a saddle-node bifurcation at *P*_*SN*_. By contrast, plant species investing in tolerance to water stress (high *χ*) need higher precipitation rates to establish, but once established persist to lower precipitation rates (blue line). Stronger seasonal forcing results in an additional effect–a period-doubling bifurcation to vegetation oscillations with a period twice as large as the annual periodicity, hereafter a 2:1 response. The instability occurs from the 1:1 solution branch at *P*_*PD*_, well before the saddle-node bifurcation at which it disappears, as Fig. 2b shows.

**Figure 3:**
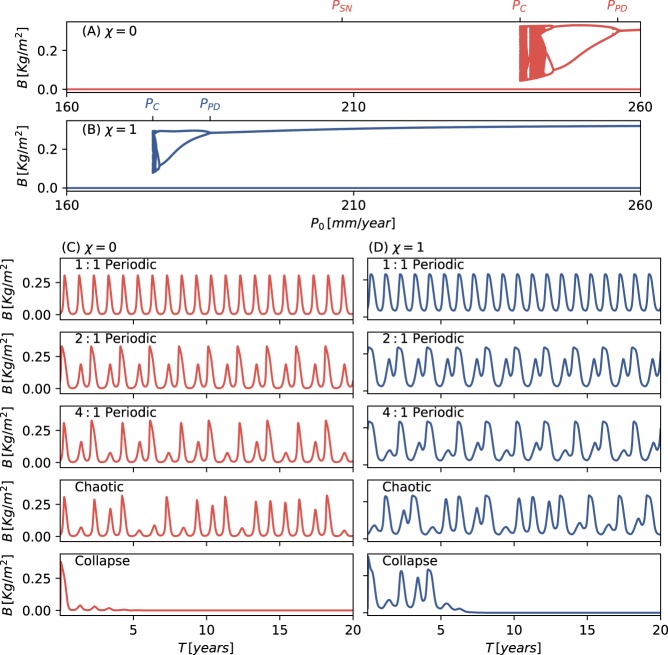
Period-doubling route to chaos along the rainfall gradient and early collapse to bare soil, obtain by numerical integration of Eq. (2), for a fixed seasonality strength *a* = 1. Bifurcation diagrams (**A**,**B**) depict the annual maximal biomass values as functions of the mean annual precipitation value *P*_0_ for tradeoff parameter values of *χ* = 0 and *χ* = 1 respectively. They show cascades of period-doubling bifurcations as the mean annual precipitation *P*_0_ is decreased, followed by a range of chaotic oscillations, and collapse to bare soil at *P*_*C*_. (**C**,**D**) typical time series showing 1:1, 2:1 and 4:1 periodic oscillations, chaotic oscillations, and collapse to bare soil. Parameters are as in Table 1.

The period-doubling bifurcation is the first in a cascade of successive period-doubling bifurcations that leads to chaotic dynamics^34,35^, as the bifurcation diagrams and time series in Fig. 3 and the Lyapunov-exponent diagram in Supplementary Fig. 1 show. Most significantly, this period-doubling route to chaos culminates in an early collapse to the alternative stable bare-soil state (at *P*_0_ = *P*_*C*_), well before the saddle-node bifurcation, *P*_*SN*_, at which the 1:1 solution branch ceases to exist. Comparing different functional groups, the whole sequence of events spans a wider precipitation range for species investing in growth (low *χ*), as compared with species investing in tolerance (high *χ*), but starts significantly earlier, i.e. at higher *P*_0_. The occurrence of a period-doubling route to chaos followed by collapse to bare soil, is demonstrated here by reducing the mean annual precipitation *P*_0_ at constant seasonality strength *a* (Fig. 3). The same scenario can occur at constant *P*_0_ by increasing the seasonality strength *a*, as Supplementary Fig. 1 demonstrates.

### Period doubling as an early warning signal

**Figure 4:**
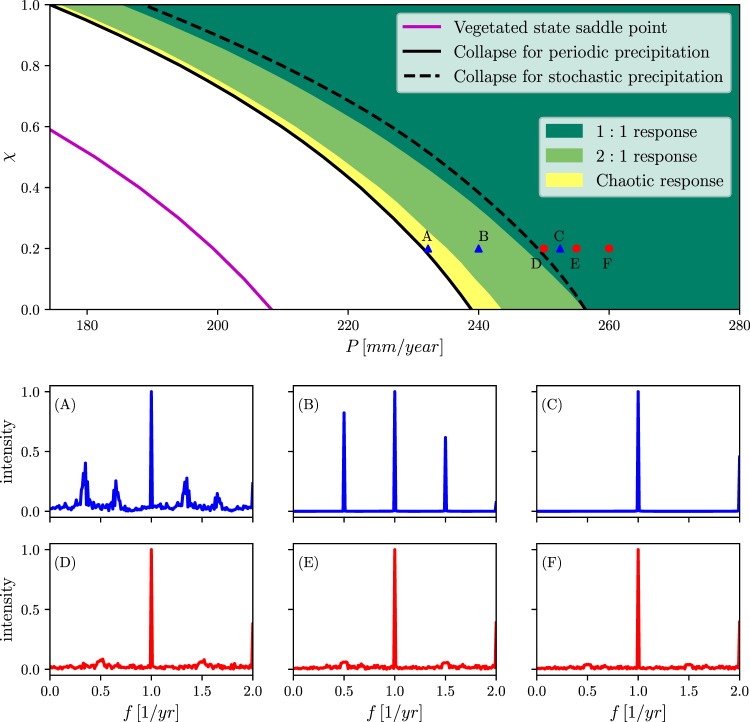
Period doubling as an early indicator for ecosystem collapse. Top panel shows the sequence of events and resulting dynamical behaviors along the precipitation axis for different functional groups (obtained from Eq. (2)), beginning with 1:1 (annual) oscillations (green), cascade of period-doubling bifurcations leading to 2*n*:1 (*n* = 1, 2, ...) oscillations (light green), chaotic oscillations (yellow), and collapse to bare soil (black solid line). Also shown is the early collapse to bare soil induced by adding a stochastic precipitation component (black dashed line). Panels A–C show power spectra (absolute values of the Fourier transform) for 1:1 periodic oscillations (**C**), 2:1 periodic oscillations (**B**) and chaotic oscillations (**A**). The precipitation rates and functional groups for which these power spectra were calculated are shown by the blue triangles in the top panel. Panels (D–F) show power spectra at increasing distances from collapse to bare soil (see red circles in top panel) for stochastic precipitation. The signatures of period doubling get smaller as the distances increase. Parameters are as in Table 1.

The period doubling route to chaos and the range of chaotic oscillations that precede the collapse to bare soil constitute a sequence of events along the rainfall gradient from which early warning signs of ecosystems at risk can be selected. These events and the ranges they occupy along the rainfall gradient for different functional groups, are shown in top panel of Fig. 4. The earliest event, as the precipitation rate *P* is decreased, is the period-doubling bifurcation from annual 1:1 oscillations (green domain) to biannual 2:1 oscillations. The 2:1 oscillations and the narrow range of additional period-doubling bifurcations occupy a fairly wide precipitation range (light-green domain) before chaotic oscillations set in (yellow domain) and collapse to bare soil occurs (black solid line). We note that this collapse takes place well before the saddle-node bifurcation or tipping point at which the vegetation state disappears (magenta line). We also draw attention to the different responses of functional groups along the tradeoff axis *χ*; groups with lower tolerance to water stress (lower *χ*) go through these processes significantly sooner, but the period-doubling and chaotic responses of these groups occupy wider precipitation ranges.

Focusing on climate extremes–severe droughts in the present case–we look for indicators that can be monitored as early as possible before the collapse point. The period-doubling bifurcation to biannual vegetation oscillations, the first event in the route to chaos, is an obvious candidate for such an indicator. Period doubling can be monitored by calculating the power spectrum of biomass oscillations and identifying the appearance of a peak at half the annual frequency, as panels B and C in Fig. 4 show. The wider period-doubling ranges of functional groups with lower *χ* (lower tolerance to water stress) further suggests a higher effectivity of this indicator for the more fragile functional groups.

The analysis so far assumed strictly periodic precipitation. Although rainfall regimes have strong annual periodicity, they also have a stochastic component^36^. Adding stochasticity to the model equations (see Methods) results in early collapse to bare soil as the black dashed line in Fig. 4 shows. As the figure shows, this line can precede the period-doubling threshold of the deterministic system, which questions the relevance of the period-doubling signature as an early indicator. However, calculations of power spectra before the collapse (red circles in Fig. 4) under conditions of stochastic precipitation do show signatures of period doubling. These results imply the applicability of period-doubling signatures in power spectra as early indicators even in stochastic systems. This should not come as a big surprise as noisy precursors of nonlinear instabilities are well known behaviors in stochastic dynamical systems^37^.

## Discussion

We studied here a vegetation model for water-limited ecosystems that includes the soil-depth dimension, by considering two soil layers, and, thereby, better captures vegetation-soil interactions than single-soil layer models. We further distinguished among species that assume different tradeoffs between investments in fast growth and in tolerance to droughts, and took into account seasonal rainfall periodicity. Three main findings were presented: (1) The appearance of a cascade of period doubling bifurcations, as the precipitation rate is decreased, which culminates in chaotic oscillations. (2) Collapse to bare soil, within the range of chaotic oscillations, well before the tipping point where the vegetation state disappears in a saddle-node bifurcation. (3) Earlier collapse, and wider ranges of period doubling and chaos, of functional groups that favor investment in fast growth. Based on these findings we proposed the detection of period-doubling signatures in power spectra as an indicator of ecosystem vulnerability to severe droughts. We further confirmed the applicability of this indicator for stochastic precipitation of moderate strength. The suggested indicator may not apply to stronger stochasticity, although signal-processing methods, such as averaging, may improve the signal-to-noise ratio. At strong stochasticity flickering behavior may set in, taking the form of back and forth switching between bare soil and vegetation oscillations. In that case the flickering itself can serve as as an early indicator of collapse^15,38^.

The first two findings directly project on the concern of ecosystem collapse by climate extremes. The finding of early collapse by chaotic oscillations, when seasonal forcing is taken into account, shifts the attention from indicators that signal the approach to a tipping point to indicators that can anticipate chaotic oscillations. The finding of a cascade of period-doubling bifurcations that precede the onset of chaos provides the preliminary processes needed to determine vulnerability to extreme events. Early warning signals based on critical slowing down can in principle probe these bifurcations, but are likely to provide false alarms, because they do not contain information about the collapse in the chaotic range further down the rainfall gradient, as the proposed indicator does.

The third finding projects on the concern of newly-formed dryland ecosystems that lack evolutionary history of water stress and, therefore, consist mostly of functional groups with poor tolerance to water stress. According to our results, such ecosystems are, not surprisingly, expected to suffer from earlier collapse to bare soil, but the ranges of period-doubled and chaotic oscillations are wider. This property of newly-formed dryland ecosystems allows for earlier warning and, thus, make the proposed indicator more effective.

To what extent should we expect the results reported here to be applicable to other ecological contexts, not necessarily associated with drylands? In particular, are there examples of ecosystems that show the same sequence of dynamical behaviors along an environmental gradient or by increasing seasonal forcing? This sequence includes ranges of annual (1:1) periodic oscillations, multi-annual periodic oscillations–predominantly biannual (2:1), chaotic oscillations, and ecosystem collapse. We have already mentioned the study of a rocky intertidal community in which chaotic oscillations were empirically found and model studies showed a transition from annual periodic oscillations to biannual periodic oscillations and further on to chaotic oscillations as the forcing strength was increased^24^. Similar behaviors have been found in seasonally forced predator-prey models, such as the Lotka-Volterra and the Rosenzweig-MacArthur models^39,40^. Thus, the first three dynamical behaviors have indeed been found in ecological contexts other than dryland vegetation. However, the fourth behavior, ecosystem collapse to a dysfunctional state, appears to be missing in these model studies. This behavior is expected to be found in the presence of feedback mechanisms that stabilize the dysfunctional state to form bistability ranges of functional and dysfunctional states. An example of a non-dryland context of this kind is coupled plankton-oxygen dynamics, where model studies show bistability of a productive phyto-zooplankton state and an extinction state^41^. Interestingly, the plankton-oxygen system also has oscillatory modes (damped or growing) in an appropriate range of environmental parameters (e.g. temperature), but the effects of seasonal forcing and, thus, the possible emergence of period doubling and chaos, have not been studied.

A common property shared by the vegetation model, the intertidal-community model, and the predator-prey models that show chaotic oscillations, is the absence of oscillations in the unforced systems, but the presence of oscillatory decay of small perturbations about stable functional states, indicating the existence of damped oscillatory modes. In the absence of these modes the response to seasonal forcing appears to be simple annual (1:1) periodic oscillations^42^, i.e. no period doubling and chaotic oscillations.

Based on these observations, we expect the proposed early indicator for ecosystem collapse–period-doubling signatures in power spectra–to apply to ecosystems that meet the following three conditions: (i) bistability ranges of functional and dysfunctional ecosystem states (in order for the question of collapse to be meaningful), (ii) sufficiently strong seasonal forcing, (iii) oscillatory modes, even damped. The bistability condition calls for negative-feedback mechanisms that stabilize the two states against small disturbances. In the context of dryland vegetation, these mechanisms may include, for example, high evaporation rate in bare soil, which acts to prevent seed germination and vegetation growth, and significantly reduced evaporation rate by shading in vegetated soil, which acts to sustain already established vegetation. In predator-prey systems, bistability can arise in systems with strong Allee effect^43–45^. Strong seasonality is realized in many dryland ecosystems as sharp rainfall and temperature variations. In predator-prey systems, strong seasonality can also be realized by sharp variations in precipitation and temperature that affect the prey’s carrying capacity and the predation success probability. Finally, the condition of oscillatory modes is generally met in consumer-resource or predator-prey systems where the growth of consumers or predators is limited by a consumable resource or a diminishing prey population^46^.

We confined ourselves in this study to temporal dynamics, excluding possible spatial variations of the state variables. In homogeneous systems, such variations can originate from two distinct mechanisms. The first is pattern-forming (Turing-like) instabilities that result in spatial self-organization. The proposed period-doubling indicator may apply to this case as well, but further studies are needed to test this extended context. Another possible mechanism for the formation of spatial patterns is associated with a multiplicity of stable states of uniform oscillations that differ in their phases of oscillation^47,48^. Multiple stable phase states of this kind are expected in precipitation ranges of period-multiplied oscillations. For example, in the range of period-doubling, we expect to find two symmetric oscillatory solutions that differ from one another by a phase shift of *π*. This is because of the invariance of the model equations to time-translations that are equal to the period of seasonal forcing (one year). The appearance of phase patterns consisting of alternating domains of different phases of oscillations may provide another indicator for sensitivity to climate extremes as it indicates the entrance to the period-doubling range. Here, too, further studies are needed to confirm these expectations.

In the Anthropocene era, where a multitude of ecological and environmental crises are reported worldwide, methodologies for the detection of early warning signals have become crucial. Considering plants that represent different stages of adaptation to dryland conditions, and their responses to strong seasonality, we are able to suggest a novel approach to detect vulnerability to ecosystem collapse, both in existing and in newly formed dryland ecosystems. The approach is based on the identification of a generic process in forced, damped oscillatory systems–period-doubling route to chaos–that precedes ecosystem collapse. Therefore, unlike early warning signals based on critical slowing down, our approach is applicable to climatic extremes occurring even far from the collapse point. We believe that our suggested approach, albeit still theoretical, provides a promising step toward the establishment of a simple detection method based on monitoring changes in vegetation biomass. As many organizations and countries accumulate such data in the last years, we believe that adopting the approach is feasible. Because of the generic nature of period-doubling route to chaos in forced, damped oscillatory systems, we expect our approach to be applicable to a variety of ecological contexts other than dryland ecosystems.

## Methods

### Model equations

Our model is based on an earlier two-soil-layers model introduced by Baudena *et al*.^27^. We simplified Baudena *et al*. model by making two main assumptions. The first is the consideration of plant species with confined root systems. This assumption allows us to integrate the root-kernel integrals and to obtain simpler algebraic forms. The second assumption is the consideration of soil types characterized by high infiltration rates, such as sandy soil. The fast infiltration of surface water into the soil prevents overland water flows and allows the elimination of the surface-water equation. For further information about this model simplification, the reader is referred to the Supplementary Information in ref. ^49^. In addition to these simplifications, we restrict ourselves in this study to plant species that are incapable of self-organizing in spatial patterns, assuming weak pattern-forming feedbacks, which allows us to assume spatially uniform states and to drop the spatial-derivative terms. Another simplification yet is the elimination of the terms in the model of Baudena *et al*.^27^ that handle the effect of over-saturation of the two soil layers (the Ω functions), assuming over-saturation is negligible.

Following these simplifications, our model consists of the following ordinary differential equations for the biomass density, *B*, the top-soil water variable, *s*_1_, and the deep-soil water variable, *s*_2_:

2a $$dB/dt=\Lambda B(1-\frac{B}{K}){(1+EB)}^{2}n{Z}_{2}{s}_{2}-M({s}_{2})B$$

2b $$n{Z}_{1}d{s}_{1}/dt=P-\frac{n{Z}_{1}N}{1+R\frac{B}{K}}{s}_{1}-{K}_{s}{s}_{1}^{c}$$

2c $$n{Z}_{2}d{s}_{2}/dt={K}_{s}{s}_{1}^{c}-{K}_{s}{s}_{2}^{c}-\Gamma B{(1+EB)}^{2}n{Z}_{2}{s}_{2},$$

where *t* is time.

We refer the reader to Table 1 for explanations of the model parameters, their numerical values, and their units. Here, we add explanations about the various terms that appear in these equations. The overall biomass growth rate is affected by water availability (the factor *nZ*_2_*s*_2_), the root branching and extension in the vertical direction (the factor (1 + *EB*)^2^) and by late-growth limitation, such as self-shading (the factor 1 − *B*/*K*). We verified that the same qualitative results and conclusions apply to more realistic water dependencies of the biomass growth rate, such as a Monod dependence^50^.

**Table 1:** A list of all quantities in the model equations, their meaning, units and their numerical values.

Quantity	Description	Units	Value/range
*B*	Biomass density	*kg*/*m*^2^	—
*s*_1_	Upper soil layer moisture content	—	[0, 1]
*s*_2_	Lower soil layer moisture content	—	[0, 1]
*P*_0_	Mean annual precipitation rate	*mm*/*y*	[0, 1000]
*a*	Precipitation modulation amplitude (seasonality strength)	—	[0, 1]
*Z*_1_	Depth of the upper soil layer	*mm*	100
*Z*_2_	Depth of the lower soil layer	*mm*	990
*n*	Soil porosity	—	0.5
*K*_*s*_	Saturated hydraulic conductivity	*m*/*y*	2 × 10^4^
*c*	Leakage exponent	—	2
*N*	Soil water evaporation rate	*y*^−1^	100
Λ_*min*_, Λ_*max*_	Minimal and maximal biomass growth rate per unit soil water	(*kg*/*m*^2^)^−1^*y*^−1^	0.35, 0.4
Γ	Soil water consumption rate per unit biomass	(*kg*/*m*^2^)^−1^*y*^−1^	4.0
*K*	Maximum standing biomass	*kg*/*m*^2^	0.4
*E*	Root’s augmentation per unit biomass	(*kg*/*m*^2^)^−1^	4
*M*_0_	Rate of biomass loss due to mortality	*y*^−1^	20
*M*_*s*,*min*_, *M*_*s*,*max*_	Minimal and maximal attenuation of the mortality rate	*y*^−1^	0, 8
*R*	Evaporation reduction due to shading	—	2.0
${s}_{2}^{\ast }$	Threshold for activation of survival mechanism	—	0.07
*α*	Sigmoid function shape parameter	—	100
*χ*	Tradeoff parameter	—	[0, 1]
*β*	Community structure parameter	—	1
*P*_*s*_	Mean annual precipitation rate for Gamma distribution	*mm*/*y*	[0, 1000]
*σ*	The scale parameter of the inter-annual	*mm*/*y*	[0, 100]

The function *M* = *M*(*s*_2_) represents the biomass decay rate and is given by

3 $$M={M}_{0}-{M}_{s}{[1+{e}^{\alpha ({s}_{2}-{s}_{2}^{\ast })}]}^{-1}\mathrm{}.$$

This form represents the activation of a survival mechanism as the deep-soil moisture, *s*_2_, drops below a characteristic value, ${s}_{2}^{\ast }$, which results in a reduced mortality rate from values approaching *M*_0_ at high *s*_2_ to values approaching *M*_0_ − *M*_*s*_ at low *s*_2_.

Water in the top soil is increased by time-dependent precipitation *P*(*t*), and decreased by evaporation (the term $\frac{n{Z}_{1}N}{1+R\frac{B}{K}}{s}_{1}$) and by percolation to the deep soil layer (the term ${K}_{s}{s}_{1}^{c}$). The evaporation rate in the presence of vegetation is reduced by shading, an effect that is quantified by the parameter *R*. Finally, water in the bottom layer is increased by percolation of water from the top layer (the term ${K}_{s}{s}_{1}^{c}$) and is decreased by two processes: further percolation to yet deeper soil that is beyond the reach of the plants’ roots (the term ${K}_{s}{s}_{2}^{c}$), and water uptake by the plants’ roots (the term Γ*B*(1 + *EB*)^2^*nZ*_2_*s*_2_). The parameter *K*_*s*_ is the saturated soil conductivity, and *c* is a parameter related to the pore size distribution of the soil^51,52^.

### Species pool

We consider a pool of species in the form of functional groups that share similar values of two functional traits, the plant-growth rate Λ and the attenuation of the mortality rate *M*_*s*_ (see Eq. (3)). We further assume a tradeoff between these functional traits and introduce a dimensionless tradeoff parameter 0 < *χ* < 1 in terms of which the two functional traits are given by:

4a $$\Lambda ={\Lambda }_{max}+{\chi }^{\beta }({\Lambda }_{min}-{\Lambda }_{max})$$

4b $${M}_{s}={M}_{s,max}+{\mathrm{(1}-\chi )}^{\beta }({M}_{s,min}-{M}_{s,max})\,\mathrm{}.$$

The parameter *β* controls the form of the tradeoff: linear for *β* = 1, convex for *β* > 1 and concave for *β* < 1, as Supplementary Fig. 2 shows. According to Eq. (4) low *χ* values correspond to species investing mostly in fast growth, whereas high *χ* values correspond to species investing mostly in survival mechanisms.

### Seasonal forcing

The seasonal forcing is capture by assuming a time-dependent precipitation *P*(*t*) as given by Eq. (1). We consider this form with constant mean annual rainfall *P*_0_ to describe periodic rainfall with amplitude *a* and period of one year, or with *P*_0_ taken from a Gamma distribution of Pearson type III^53,54^:

5 $${P}_{0} \sim {\rm{Gamma}}(k={P}_{s}/\sigma ,\mu ={P}_{s}),$$

to describe stochastic rainfall, where *P*_*s*_ is the inter-annual mean precipitation, and *σ* is standard deviation of the distribution.

### Numerical calculations

The time integration of the system of ordinary differential Eq. (2) has been carried out using both the Runge-Kutta method and an implicit method based on backward-differentiation formulas. The bifurcation diagrams Fig. 2 have been obtained using the AUTO-07P^55^ continuation software. The stability information was derived either from the eigenvalues of the numerical Jacobian for the unforced system or by the Floquet multipliers for the periodically forced system. The bifurcation diagrams in Fig. 3A,B were obtained by integrating the model equations for fixed values of *P*_0_ in the ranges displayed in the diagrams, and plotting the biomass values that correspond to maxima of the time-series. We have used the Fast Fourier transform package of Python’s SciPy software to obtain the power spectra in Fig. 4.

## Supplementary information

Supplementary Information
