# Supplementary material for: Period doubling as an indicator for ecosystem sensitivity to climate extremes
Source: Sci Rep. 2019 Dec 20;9:19577. doi: 10.1038/s41598-019-56080-z (PMC6925204; doi:10.1038/s41598-019-56080-z)
Supplement: Supplementary file 1 — Supplementary Information [file 41598_2019_56080_MOESM1_ESM.pdf]

# Supplementary material for: Period doubling as an indicator for ecosystem sensitivity to climate extremes

Omer Tzuk[1], Sangeeta Rani Ujjwal[2], Cristian Fernandez-Oto[2,3], Merav Seifan[4] & Ehud Meron[1,2]

[1]*Department of Physics, Ben-Gurion University, Beer Sheva, 84105, Israel*
[2]*Department of Solar Energy and Environmental Physics, Blaustein Institutes for Desert Research, Ben-Gurion University of the Negev, Sede Boqer Campus 84990, Israel*
[3]*Complex Systems Group, Facultad de Ingeniería y Ciencias Aplicadas, Universidad de los Andes, Av. Mon. Alvaro del Portillo 12.455, Santiago, Chile*
[4]*Mitrani Department of Desert Ecology, SIDEER, Blaustein Institutes for Desert Research, Ben-Gurion University of the Negev, Sede Boqer Campus 84990, Israel*

**Extra figures**

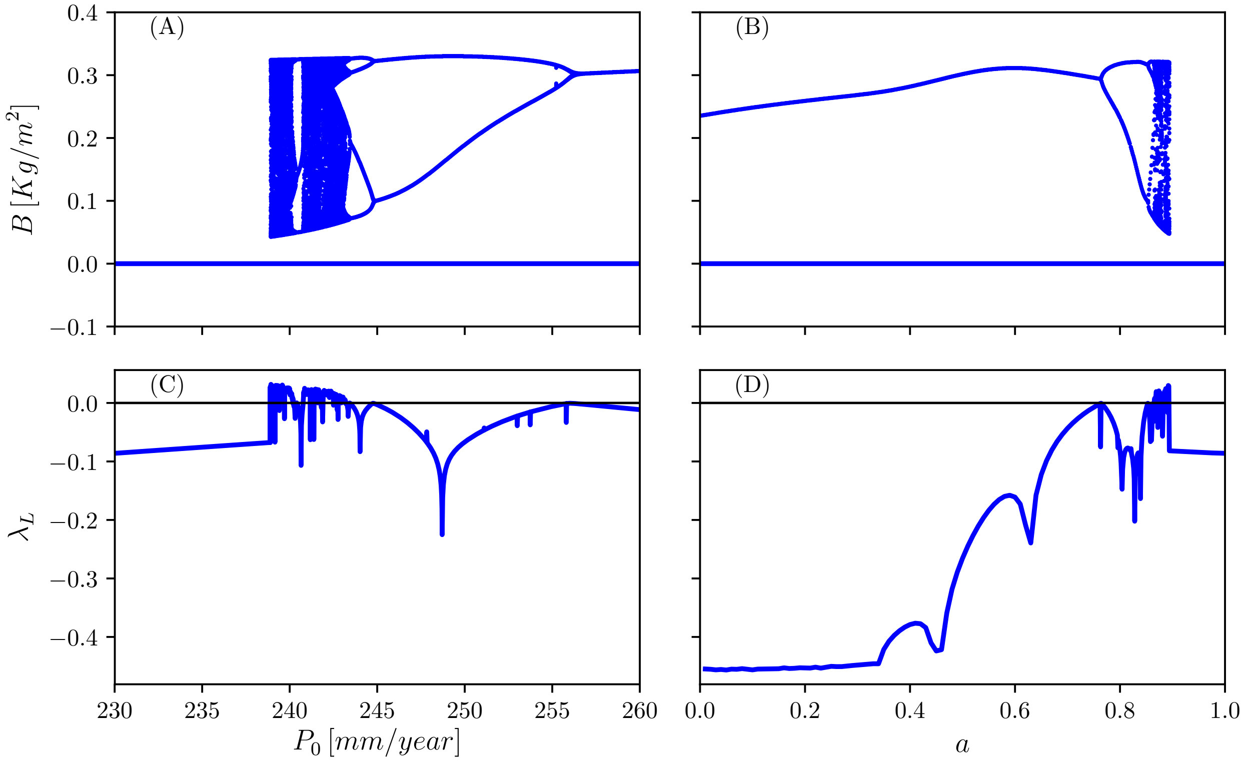


Figure 1: Bifurcation diagrams showing the period-doubling route to chaos in Eqs. (2) of main text. The route to chaos is initiated by a period-doubling bifurcation occurring as the mean annual precipitation rate $P_0$ is decreased (A) or as the seasonality strength $a$ is increased (B). The vertical axes in (A) and (B) show the above-ground biomass density. Note the existence of a stable bare-soil solution ($B = 0$) throughout the entire parameter range depicted. Ranges of positive Lyapunov exponents, $\lambda_L$, indicating chaotic oscillations along the precipitation and seasonality-strength axes are shown in panels (C) and (D).

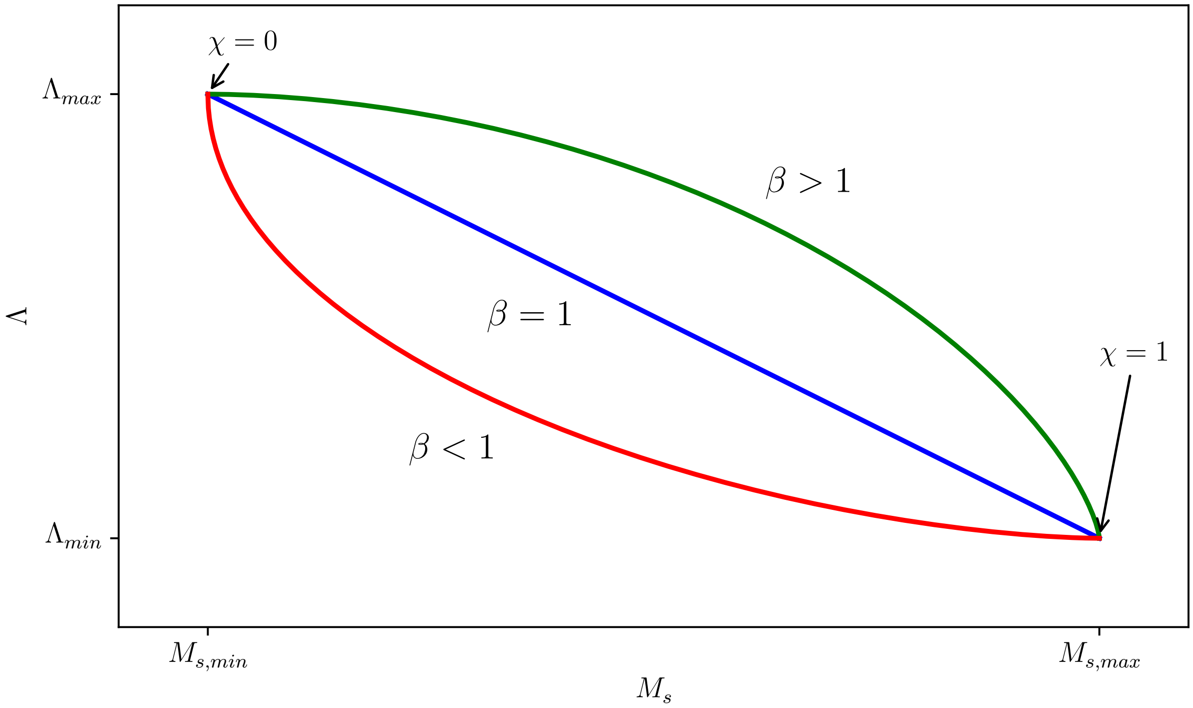


Figure 2: A graphic presentation of the tradeoff defined by Eqs. (4) of main text. Depending on the value of the exponent $\beta$, the tradeoff is linear ($\beta = 1$, blue line), convex ($\beta > 1$, green line), or concave ($\beta < 1$, red line).
